# Non-metal-templated approaches to bis(borane) derivatives of macrocyclic dibridgehead diphosphines via alkene metathesis

**DOI:** 10.3762/bjoc.14.211

**Published:** 2018-09-07

**Authors:** Tobias Fiedler, Michał Barbasiewicz, Michael Stollenz, John A Gladysz

**Affiliations:** 1 Department of Chemistry, Texas A&M University, PO Box 30012, College Station, Texas 77842-3012, USA; 2 Institut für Organische Chemie and Interdisciplinary Center for Molecular Materials, Friedrich-Alexander-Universität Erlangen-Nürnberg, Henkestraße 42, 91054 Erlangen, Germany; 3 Present address: Faculty of Chemistry, University of Warsaw, Pasteura 1, 02-093 Warszawa, Poland; 4 Present address: Department of Chemistry and Biochemistry, Kennesaw State University, 370 Paulding Building NW, MD#1203, Kennesaw, Georgia 30144, USA

**Keywords:** alkene metathesis, crystal structures, homeomorphic isomerization, hydrogenation, *in*/*out* isomers, metathesis, phosphine boranes

## Abstract

Two routes to the title compounds are evaluated. First, a ca. 0.01 M CH_2_Cl_2_ solution of H_3_B·P((CH_2_)_6_CH=CH_2_)_3_ (**1**·BH_3_) is treated with 5 mol % of Grubbs' first generation catalyst (0 °C to reflux), followed by H_2_ (5 bar) and Wilkinson's catalyst (55 °C). Column chromatography affords H_3_B·P(*n-*C_8_H_17_)_3_ (1%), H_3_B·*P*((CH_2_)_13_*C*H_2_)(*n*-C_8_H_17_) (8%; see text for tie bars that indicate additional phosphorus–carbon linkages, which are coded in the abstract with italics), H_3_B·*P*((CH_2_)_13_*C*H_2_)((CH_2_)_14_)*P*((CH_2_)_13_*C*H_2_)·BH_3_ (**6**·2BH_3_, 10%), *in,out*-H_3_B·P((CH_2_)_14_)_3_P·BH_3_ (*in,out*-**2**·2BH_3_, 4%) and the stereoisomer (*in,in*/*out,out*)-**2**·2BH_3_ (2%). Four of these structures are verified by independent syntheses. Second, 1,14-tetradecanedioic acid is converted (reduction, bromination, Arbuzov reaction, LiAlH_4_) to H_2_P((CH_2_)_14_)PH_2_ (**10**; 76% overall yield). The reaction with H_3_B·SMe_2_ gives **10**·2BH_3_, which is treated with *n*-BuLi (4.4 equiv) and Br(CH_2_)_6_CH=CH_2_ (4.0 equiv) to afford the tetraalkenyl precursor (H_2_C=CH(CH_2_)_6_)_2_(H_3_B)P((CH_2_)_14_)P(BH_3_)((CH_2_)_6_CH=CH_2_)_2_ (**11**·2BH_3_; 18%). Alternative approaches to **11**·2BH_3_ (e.g., via **11**) were unsuccessful. An analogous metathesis/hydrogenation/chromatography sequence with **11**·2BH_3_ (0.0010 M in CH_2_Cl_2_) gives **6**·2BH_3_ (5%), *in,out*-**2**·2BH_3_ (6%), and (*in,in*/*out,out*)-**2**·2BH_3_ (7%). Despite the doubled yield of **2**·2BH_3_, the longer synthesis of **11**·2BH_3_ vs **1**·BH_3_ renders the two routes a toss-up; neither compares favorably with precious metal templated syntheses.

## Introduction

**Scheme 1:**
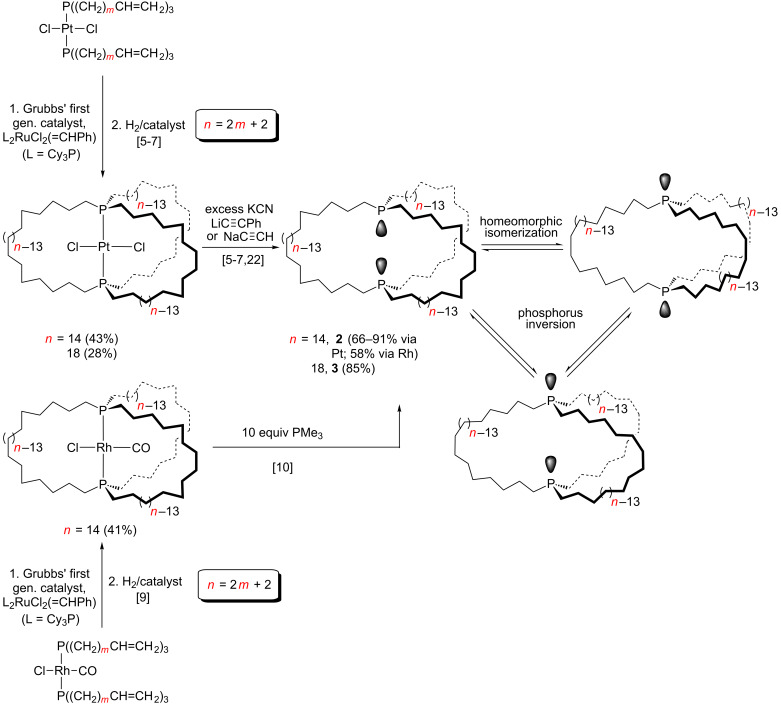
Syntheses of gyroscope like platinum and rhodium complexes and dibridgehead diphosphines derived therefrom.

We have found that a variety of metal complexes with *trans*-phosphine ligands of the formula P((CH_2_)*_m_*CH=CH_2_)_3_ (**1**; *m* = 4–14) undergo threefold interligand ring closing alkene metatheses to give, after hydrogenations, metal complexes of *in*,*in* isomers of macrocyclic dibridgehead diphosphines [1–13]. Representative examples with square planar complexes are shown in Scheme 1. Analogous sequences with trigonal bipyramidal substrates proceed in somewhat higher overall yields, as analyzed elsewhere [1–4]. Setaka has developed a similar chemistry in which the phosphorus atoms are replaced by silicon and the metal fragment by *p*-phenylene (*p*-C_6_H_4_) or related aromatic moieties [14–19]. These types of compounds are viewed as promising candidates for molecular gyroscopes [14–21].

We subsequently developed an interest in the free dibridgehead diphosphine ligands P((CH_2_)*_n_*)_3_P (*n* = 14, **2**; 18, **3**), prompted in part by the unexpected discovery of the facile demetalations shown in Scheme 1 [5–6,10,22]. Such compounds were previously known only for much smaller ring sizes (*n* < 4) [23]. These reactions require excesses of certain nucleophiles, and the mechanisms remain under study. The yields are quite good, but the routes are stoichiometric in precious metals. Although the metals can be recovered as species such as K_2_Pt(CN)_4_ or RhCl(PMe_3_)_3_, we have nonetheless sought to develop more economical protocols.

The analogous Fe(CO)_3_ adducts are easily prepared [1–4], but in efforts to date it has not been possible to efficiently remove the dibridgehead diphosphine ligands from the low cost iron fragment. Oxidations that lead to the corresponding dibridgehead diphosphine dioxides (O=)P((CH_2_)*_n_*)_3_P(=O) have exhibited promise, but purification has been problematic [24]. Indeed, phosphine oxides are everyday precursors to phosphines, so we have considered various non-metal-templated routes to **2·**2(=O), **3**·2(=O), and related species. However, as described in the discussion section, the yields have not been competitive [25].

Another preliminary point concerns the ability of macrocyclic dibridgehead diphosphorus compounds to exhibit *in*/*out* isomerism [26]. As shown in Scheme 1, there are three limiting configurations for **2** and **3**: *in*,*in*, *out*,*out*, and *in*,*out* (identical to *out*,*in*). The first two, as well as the degenerate *in*,*out* pair, can rapidly interconvert by a process termed homeomorphic isomerization [26–27], which is akin to turning the molecules inside out. Readers are referred to earlier publications in this series for additional details [22,25,28–30]. Interconversions between the *in*,*in*/*out*,*out* and *in*,*out*/*out*,*in* manifolds require phosphorus inversion and temperatures considerably in excess of 100 °C.

**Scheme 2:**
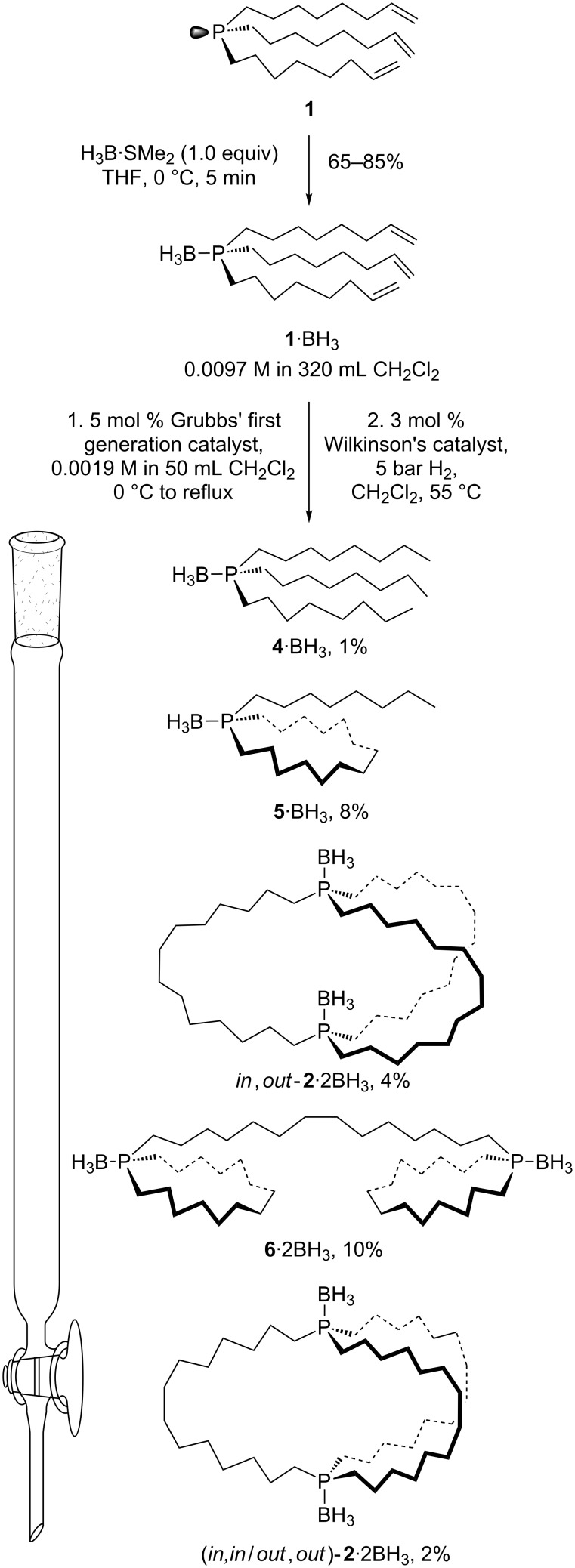
Synthesis and alkene metathesis of the monophosphorus precursor **1**·BH_3_.

In this paper, we describe two non-metal-templated approaches to **2** that are based upon metatheses of phosphine boranes of alkene containing phosphines. The first involves the monophosphorus precursor H_3_B·P((CH_2_)_6_CH=CH_2_)_3_ (**1**·BH_3_) [31], and the second a diphosphorus precursor in which one of the methylene chains linking the two phosphorus atoms has already been installed. The advantages and limitations of each are analyzed in detail. Some of the results (Scheme 2) have appeared in the supporting information of a preliminary communication [28], and others in a dissertation [32].

## Results

### 1. Monophosphorus precursors

As reported earlier [31], the alkene containing phosphine P((CH_2_)_6_CH=CH_2_)_3_ (**1**) can be prepared in 87% yield from the reaction of PCl_3_ and MgBr(CH_2_)_6_CH=CH_2_. Following the addition of H_3_B·SMe_2_, the phosphine borane **1**·BH_3_ can be isolated in 65–85% yields [31], as shown in Scheme 2. It is critical to avoid an excess of H_3_B·SMe_2_, as this brings the C=C units into play. In fact, when substoichiometric amounts of H_3_B·SMe_2_ are added to THF solutions of purified **1**·BH_3_, gels immediately form.

A ca. 0.01 M CH_2_Cl_2_ solution of **1**·BH_3_ and a ca. 0.002 M CH_2_Cl_2_ solution of Grubbs' first generation catalyst (3 mol %) were combined at 0 °C. The mixture was warmed to room temperature, and a second charge of Grubbs' catalyst added (2 mol %). The sample was refluxed, and then filtered through silica gel. The filtrate was concentrated and treated with H_2_ (5 bar) and Wilkinson's catalyst (55 °C). The mixture was taken to dryness and the residue tediously chromatographed on a silica gel column. Numerous fractions were collected and analyzed by TLC. The mass recovery from the column was 33% of theory (for complete metathesis).

More than ten mobile products could be discerned, but only five could be isolated in pure form and ultimately identified. These are described in order of elution. Each was analyzed by NMR (^1^H, ^31^P{^1^H}, ^13^C{^1^H}; always CDCl_3_) and IR spectroscopy, mass spectrometry, and microanalysis, as summarized in the experimental section. The ^13^C{^1^H} NMR spectra proved to be most diagnostic of structure, and were analyzed in detail. The ^31^P{^1^H} NMR spectra were all very similar (broad apparent doublets due to phosphorus boron coupling).

First, traces of a colorless oil were obtained. The ^1^H NMR spectrum showed a characteristic triplet at 0.83 ppm consistent with a terminal methyl group. The ^13^C{^1^H} NMR spectrum exhibited eight signals, two of which were phosphorus coupled doublets. One of the singlets (14.0 ppm) was typical of a terminal methyl group. Based upon these data, and the integration of the ^1^H NMR spectrum, the oil was assigned as the hydrogenated phosphine borane H_3_B·P(*n-*C_8_H_17_)_3_ (**4**·BH_3_), a known compound [33]. The yield was only 1%.

Next, another colorless oil eluted. The ^1^H NMR and ^13^C{^1^H} NMR spectra again exhibited signals characteristic of a methyl group (0.86 ppm, t; 14.0 ppm, s). Integration of the ^1^H NMR spectrum established a 14:1 area ratio for the methylene (1.62–1.19 ppm) and methyl signals. The ^13^C{^1^H} NMR spectrum featured one set of seven signals and another set of eight with an intensity ratio of approximately 2:1. The less intense set resembled the signals arising from the *n*-octyl groups in **4**·BH_3_. The more intense set was very similar to the signals arising from the cyclic 
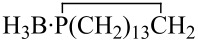
 substructures of **6**·2BH_3_ (described below) and a phosphine borane 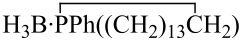 reported earlier [34]. The mass spectrum exhibited an intense ion at *m*/*z* 340 (**5**^+^, 93%), and no ions of higher mass. Hence, the oil was assigned as the monocyclic intramolecular metathesis product 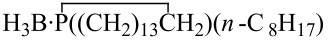
(**5**·BH_3_; see Scheme 2). The yield was 8%.

**Figure 1:**
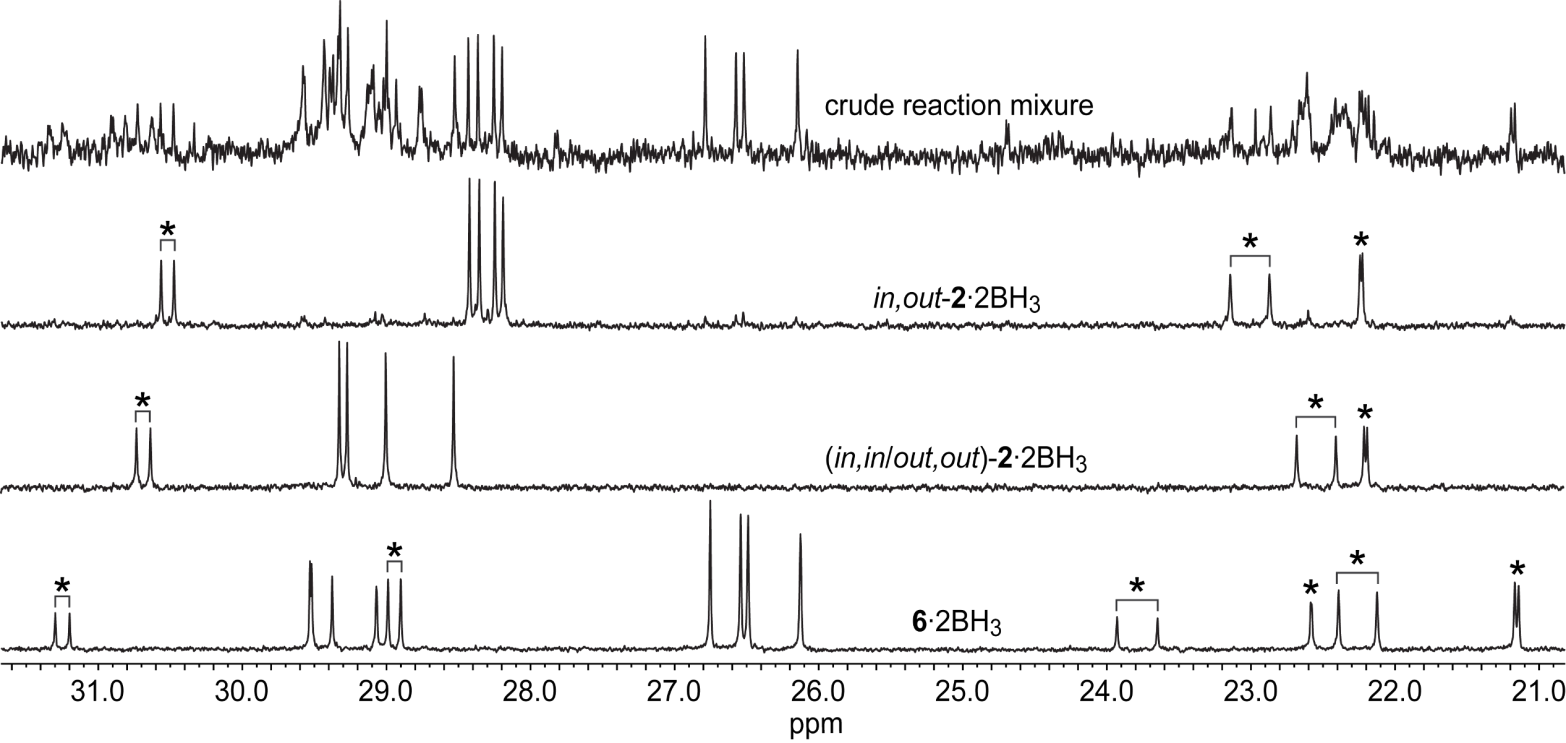
The ^13^C{^1^H} NMR spectra (CDCl_3_, 100 MHz) of *in*,*out*-**2**·2BH_3_, (*in,in*/*out*,*out*)-**2**·2BH_3_, **6**·2BH_3_, and the crude reaction mixture after hydrogenation from Scheme 5 (top); doublets are marked with an asterisk.

The third product was also a colorless oil. The ^13^C{^1^H} NMR spectrum exhibited seven signals, three of which were phosphorus coupled doublets (second spectrum from top, Figure 1). Analogous coupling patterns are found with the free dibridgehead diphosphines **2** and **3** in Scheme 1. No NMR signals diagnostic of methyl groups were present, and further analysis is presented along with that for an isomer below.

A white powder was obtained next. The ^13^C{^1^H} NMR spectrum exhibited fourteen signals, half of which were approximately twice as intense as the others. Two signals of each set exhibited phosphorus coupling. The overall pattern was quite similar to those shown by metal complexes with *cis* or *trans* coordinating diphosphine ligands of the formula 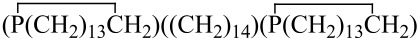 (**6**) [6–7,12–13,35]. This suggested the diphosphine diborane structure **6**·2BH_3_ (see Scheme 2), which is derived from one metathesis involving alkenyl moieties on different phosphorus atoms, and two metatheses of alkenyl moieties on identical phosphorus atoms. The yield was 10%. The structure has been confirmed by an independent synthesis (detachment of the diphosphine from a platinum complex followed by borane addition) and a crystal structure [6].

Finally, another white powder was obtained. As with the previous oil isolated above, the ^13^C{^1^H} NMR spectrum exhibited seven signals, three of which were phosphorus coupled doublets (third spectrum from top, Figure 1). Both spectra were consistent with dibridgehead diphosphine diboranes H_3_B·P((CH_2_)_14_)_3_P·BH_3_ (**2**·2BH_3_) derived from threefold intermolecular metatheses of **1**·BH_3_. Based upon independent syntheses from the dibridgehead diphosphines **2** obtained in Scheme 1 [6], they were assigned as *in,out*-**2**·2BH_3_ (4%) and the stereoisomer (*in,in*/*out,out*)-**2**·2BH_3_ (2%), as shown in Scheme 2. The depiction of the latter as an *out*,*out* (vs *in*,*in*) isomer in Scheme 2 is arbitrary, but represents the form found in a confirming crystal structure [6].

Parallel reactions were conducted with Grubbs' second generation catalyst and the nitro-Grela catalyst [36]. However, the combined yields of **2** diminished.

### 2. Diphosphorus precursors

**Scheme 3:**
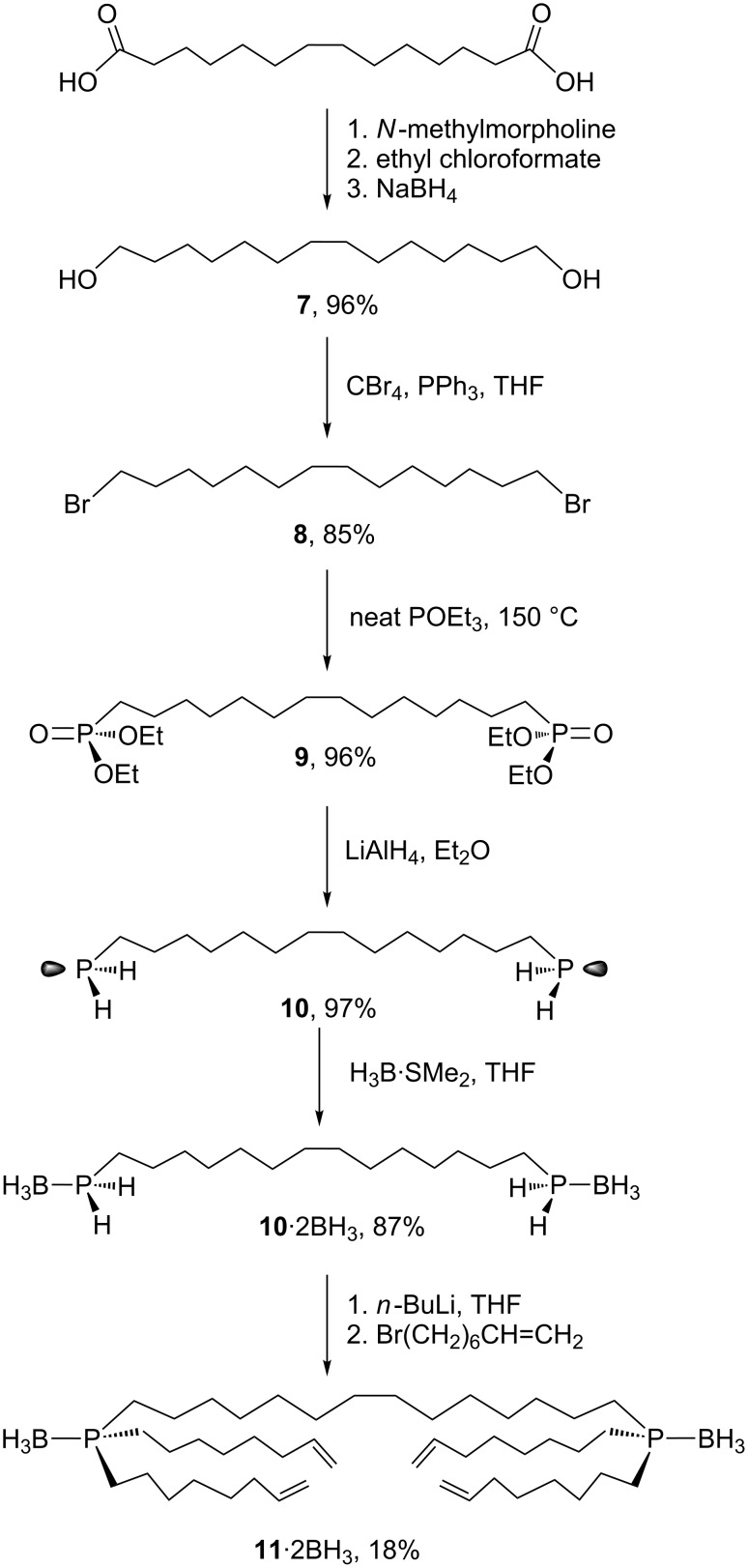
Synthesis of the diphosphorus precursor **11**·2BH_3_.

Since the yields of the cage like diphosphine diboranes **2**·2BH_3_ in Scheme 2 were – as expected – very low, alternative strategies were considered. The poor mass balance was attributed, at least in part, to the formation of oligomeric products that were retained on the column. Improvements might be expected from precursors in which one of the methylene chains tethering the two phosphorus atoms was pre-formed. Thus, we set out to prepare a tetraalkenyl metathesis precursor as shown in Scheme 3.

The first step, a previously reported reduction of commercial 1,14-tetradecanedioic acid to 1,14-tetradecanediol (**7**) [37], was followed by an Appel reaction to give 1,14-dibromotetradecane (**8**) [38–43]. An Arbuzov reaction then afforded the diphosphonate (EtO)_2_(O=)P((CH_2_)_14_)P(=O)(OEt)_2_ (**9**) [44]. Subsequent reduction with LiAlH_4_ gave the diprimary diphosphine H_2_P((CH_2_)_14_)PH_2_ (**10**) in 76% yield from **7** as a foul smelling white powder.

It has been shown that borane adducts of primary phosphines can be doubly deprotonated, and that the resulting phosphorus dianions can be bis(alkylated) [45–47]. Thus, the diphosphine **10** and H_3_B·SMe_2_ were reacted to give the diphosphine diborane H_2_(H_3_B)P((CH_2_)_14_)P(BH_3_)H_2_ (**10**·2BH_3_) as a white solid in 87% yield. A subsequent reaction with *n*-BuLi (4.4 equiv) and Br(CH_2_)_6_CH=CH_2_ (4.0 equiv) gave the tetraalkenyl target (H_2_C=CH(CH_2_)_6_)_2_(H_3_B)P((CH_2_)_14_)P(BH_3_)((CH_2_)_6_CH=CH_2_)_2_ (**11**·2BH_3_), but in only 18% yield.

**Scheme 4:**
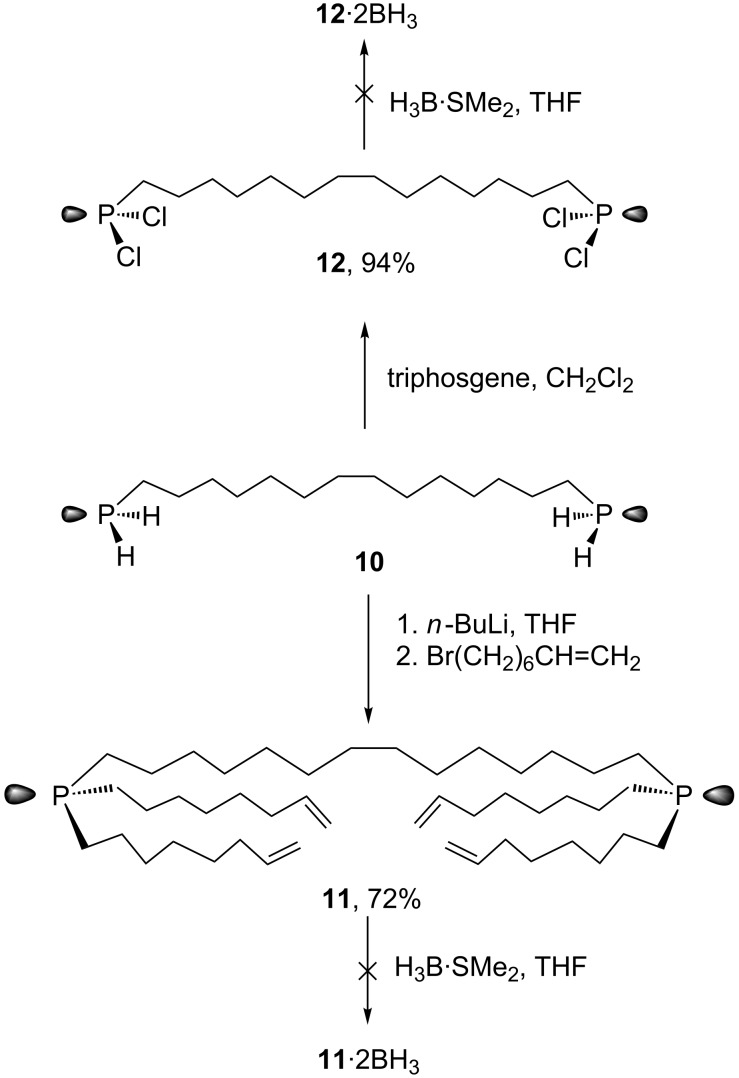
Truncated approaches to the diphosphorus precursor **11**·2BH_3_ from **10**.

Accordingly, two alternative routes to **11**·2BH_3_ were considered. The initial step for the first is depicted in Scheme 4. Primary phosphines can be doubly deprotonated, analogously to borane adducts, and the phosphorus dianions subsequently bis(alkylated) [34,48]. Thus, **10** was treated with *n*-BuLi (4.1 equiv) and then Br(CH_2_)_6_CH=CH_2_ (4.0 equiv). Work-up gave the target compound (H_2_C=CH(CH_2_)_6_)_2_P((CH_2_)_14_)P((CH_2_)_6_CH=CH_2_)_2_ (**11**) in 72% yield. However, all attempts to convert **11** to **11**·2BH_3_ gave only traces of the latter. Mainly insoluble material formed, which was presumed to be oligomeric and possibly derived from B–H additions to the alkenyl groups.

In the second approach, **10** was first converted to the tetrachloride Cl_2_P((CH_2_)_14_)PCl_2_ (**12**) in 94% yield using triphosgene, a standard reagent for the chlorination of phosphorus–hydrogen bonds [49]. Since a direct reaction with an excess of the Grignard reagent BrMg(CH_2_)_6_CH=CH_2_ would give **11**, a dead end, initial conversion to the bis(borane) adduct **12**·2BH_3_ was envisioned. However, reactions of **12** and H_3_B·SMe_2_ (2.1 equiv) afforded only insoluble material.

**Scheme 5:**
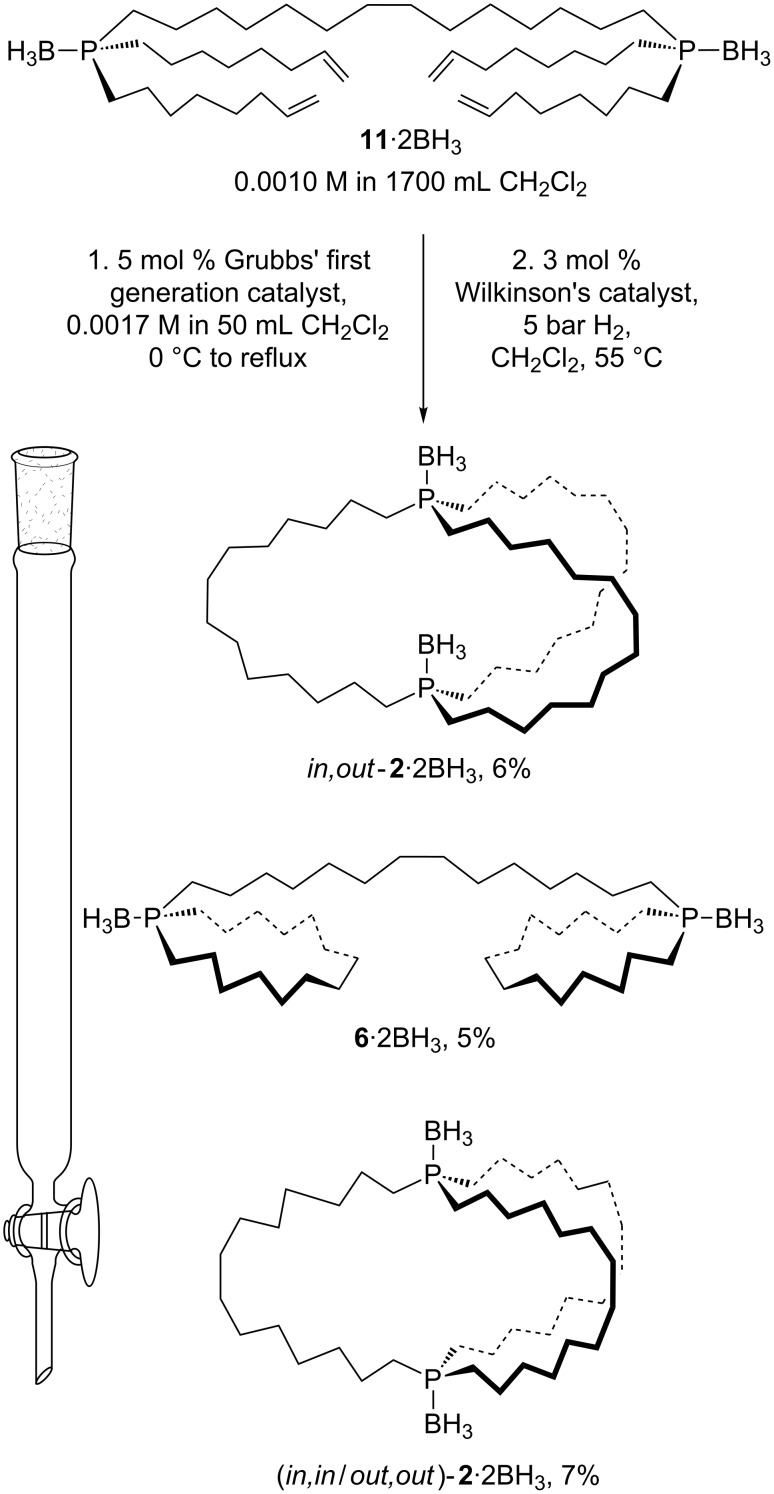
Alkene metathesis of the diphosphorus precursor **11**·2BH_3_.

Thus, despite the low yield of the final step in Scheme 3, reasonable quantities of the diphosphine diborane **11**·2BH_3_ could be stockpiled. As shown in Scheme 5, **11**·2BH_3_ was subjected to a metathesis/hydrogenation/column chromatography sequence similar to that for **1**·BH_3_ in Scheme 2. However, a tenfold higher dilution was used in the metathesis step (0.0010 M as compared to 0.010 M).

Figure 1 shows a ^13^C{^1^H} NMR spectrum of the crude product after hydrogenation stacked above spectra of the three products that could be isolated after the rather tedious column chromatography: the dibridgehead diphosphine diborane *in,out*-**2**·2BH_3_, its constitutional isomer **6**·2BH_3_, and its stereoisomer (*in,in/out,out*)-**2**·2BH_3_. It can be inferred from the top spectrum that the three products were the major components and moreover present in approximately equal amounts. However, the isolated yields were affected by the challenging separation. In particular, *in,out*-**2**·2BH_3_ and **6**·2BH_3_ eluted very closely, rendering some mixed fractions unavoidable and lowering the amounts of pure products.

Compared to the metathesis/hydrogenation sequence for **1**·BH_3_ (Scheme 2) the yields of *in,out*-**2**·2BH_3_ and (*in,in/out,out*)-**2**·2BH_3_ (Scheme 5) are higher but still poor. Taking into account the overall yields (three steps from PCl_3_ and BrMg(CH_2_)_6_CH=CH_2_ in the first synthesis vs seven steps from 1,14-tetradecanedioic acid in the second), the latter route does not offer any advantage, even if one were to improve the conversion of **10**·2BH_3_ to **11**·2BH_3_.

## Discussion

**Scheme 6:**
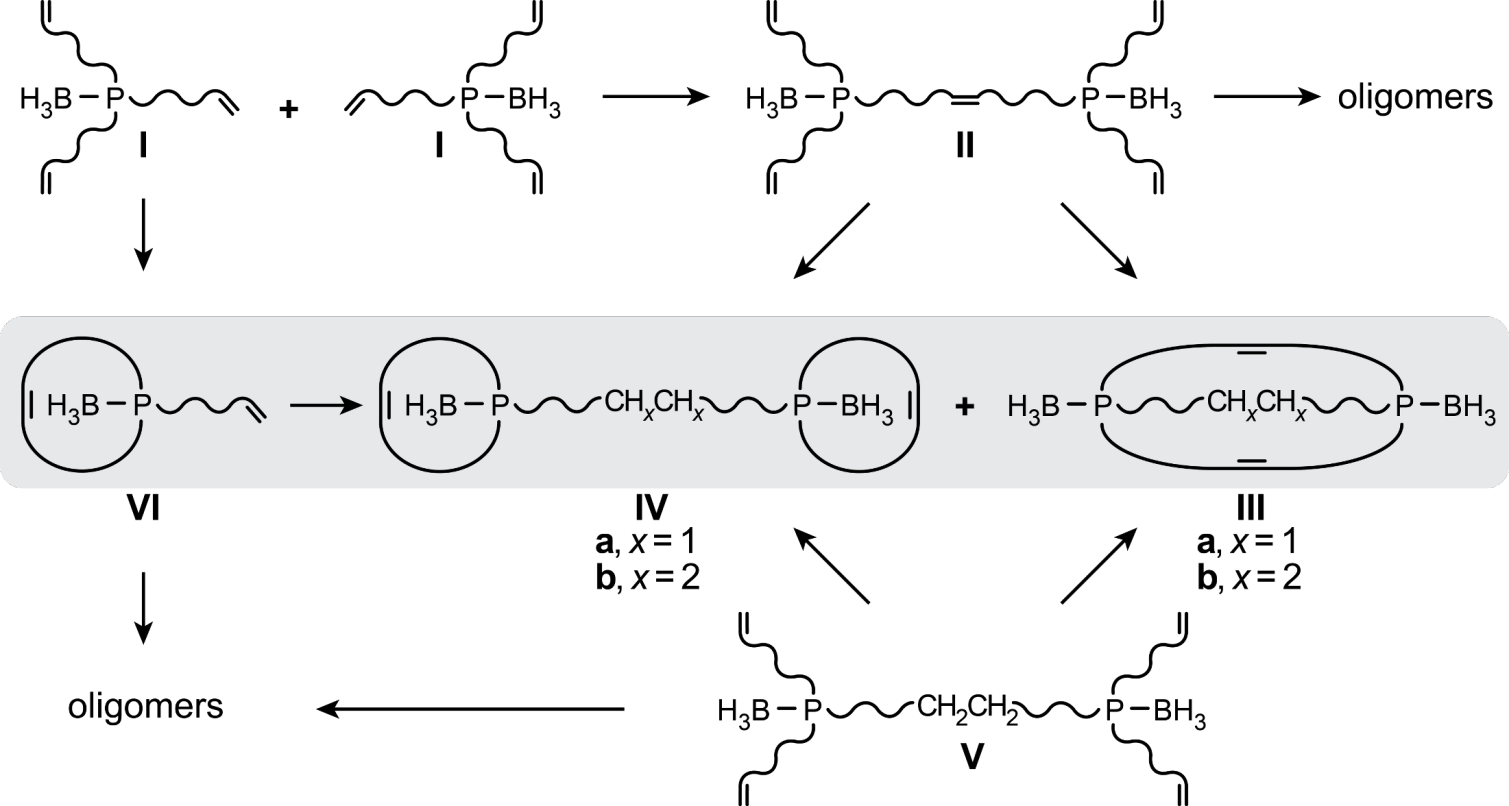
Schematic comparison of the key alkene metathesis steps in Scheme 2 and Scheme 5.

As contrasted in Scheme 6, Scheme 2 and Scheme 5 present two conceptually related routes to the isomeric title compound **2**·2BH_3_. In the first, two trialkenylphosphine boranes (**1**·BH_3_ = **I**) must undergo metathesis. The first productive step is intermolecular, giving a diphosphorus compound with a P(CH_2_)_6_CH=CH(CH_2_)_6_P tether **II** that is positioned for subsequent intramolecular ring closing steps. Those involving alkenyl groups from different phosphorus atoms are productive (leading to **2**·2BH_3_ via hydrogenation of **IIIa**), and those involving groups from the same phosphorus atoms are non-productive (leading to **6**·2BH_3_ via hydrogenation of **IVa**). In the second, the starting material has a preformed P(CH_2_)_14_P tether (**11**·2BH_3_ = **V**), and the four alkenyl groups have reactivity options (→ **IIIb** or **IVb**) analogous to those of intermediate **II** with the P(CH_2_)_6_CH=CH(CH_2_)_6_P tether. Importantly, all of these steps are presumed to be largely under kinetic control, consistent with experience with the types of metatheses in Scheme 1 [1–13,34].

Although the second route intuitively seems more favorable, after the initial intermolecular metathesis of **1**·BH_3_ (**I**), both require an equivalent series of steps to reach (after hydrogenation) **2**·2BH_3_. One reason **1**·BH_3_ is an inferior substrate is that following the initial generation of a P(CH_2_)_6_CH=Ru species, two P(CH_2_)_6_CH=CH_2_ moieties remain available for non-productive intramolecular ring closing metathesis (giving **VI**). In contrast, with the analogous intermediate derived from **11**·2BH_3_ (**V**), there is only one P(CH_2_)_6_CH=CH_2_ moiety that can give non-productive chemistry. It is also worth noting that high dilution provides less of an advantage in Scheme 2, as one wants to favor intermolecular over intramolecular metatheses in the first step. In Scheme 5, one wants to avoid intermolecular metatheses at all stages.

**Scheme 7:**
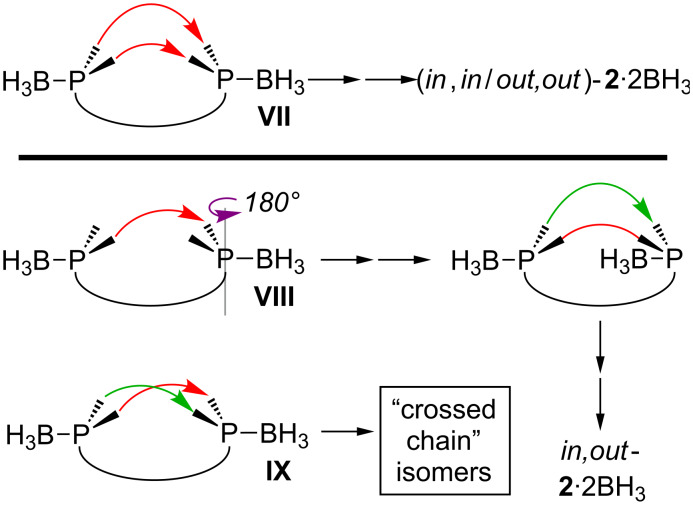
Steps that set the *in*,*in*/*out*,*out* vs *in*,*out* stereochemistry of **2**·2BH_3_ in Scheme 2 and Scheme 5.

At present, we have no rationale for the *in*,*out* vs (*in*,*in*/*out*,*out*) isomer ratios for **2**·2BH_3_. However, it is easy to map the sequence leading to each, as shown in Scheme 7. When there is only one tether between the two phosphorus atoms, the phosphorus–boron bonds can be arrayed in an *anti* fashion, as depicted in **VII**. When subsequent metatheses join alkenyl groups in the *syn* positions on each phosphorus atom (front to front and rear to rear), (*in*,*in*/*out*,*out*)-**2**·2BH_3_ must result (as drawn in Scheme 7, the *out*,*out* isomer would be the kinetic product). When the first metathesis does not join the *syn* positions, as in **VIII** (front to rear), one phosphorus–boron bond must subsequently be rotated by 180° to create a *syn* orientation for the second metathesis.

Of course, if the first metathesis step does not require a *syn* relationship (per **VIII**), the same possibility can be entertained for the second (see **IX**). This would lead to an isomeric bicyclic compound with "crossed chains". We have sought to access such species by conducting metatheses of substrates of the types in Scheme 1 that give thirty-three membered macrocycles (*n* = 30) [7]. However, none have so far been detected. Other types of crossed chain *in*/*out* isomer systems have in fact been realized [25,30].

As communicated earlier [28] and will be described more fully in a later paper, both isomers of **2**·2BH_3_ are easily deprotected to give the respective isomers of the dibridgehead diphosphine **2** in high yields. Since phosphine oxides are also easily converted to phosphines, one could consider parallel approaches to **2** via metatheses of the phosphine oxide (O=)P((CH_2_)_6_CH=CH_2_)_3_ (**1**(=O)) or diphosphine dioxide (H_2_C=CH(CH_2_)_6_)_2_(O=)P((CH_2_)_14_)P(=O)((CH_2_)_6_CH=CH_2_)_2_ (**11**·2(=O)). Given the poor results with **1**·BH_3_ in Scheme 2, no attempt has been made to explore similar reactions with **1**(=O).

**Scheme 8:**
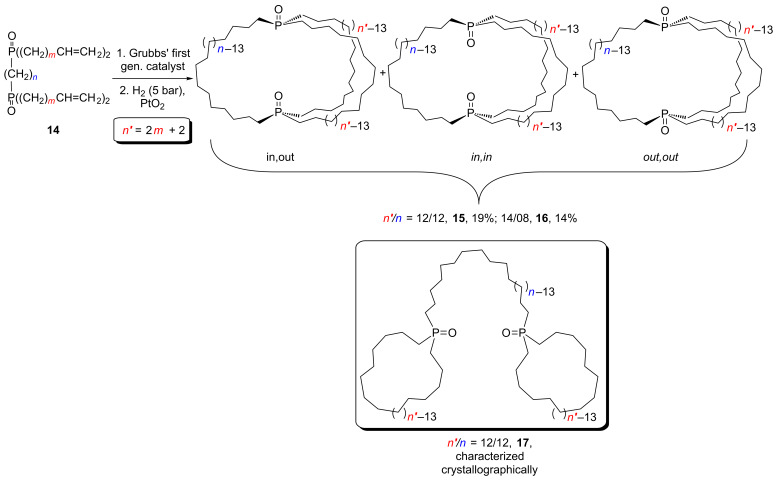
Another non-metal-templated approach to dibridgehead diphosphorus compounds.

However, as shown in Scheme 8, it has proved possible to synthesize the diphosphine dioxides **14**, in which the two phosphorus atoms are tethered by a methylene chain, in two steps in 66–68% overall yields from diethyl phosphonate ((O=)PH(OEt)_2_), Grignard reagents BrMg(CH_2_)*_m_*CH=CH_2_, base (NaH), and appropriate α,ω-dibromides Br(CH_2_)*_n_*Br [25]. Following metathesis and hydrogenation, these afford dibridgehead diphosphine oxides **15** and **16** in 14–19% yields. This is slightly better than the combined yield of *in,out*- and (*in,in*/*out,out*)-**2**·2BH_3_ in Scheme 5, although the data are not strictly comparable as the ring sizes differ. It has not yet proved possible to efficiently separate the *in*/*out* isomers of **15** and **16**. However, byproducts derived from metatheses of alkenyl groups on the same phosphorus atom – such as **17** (comparable to **6**·2BH_3_) – appear to form in much smaller amounts.

**Scheme 9:**
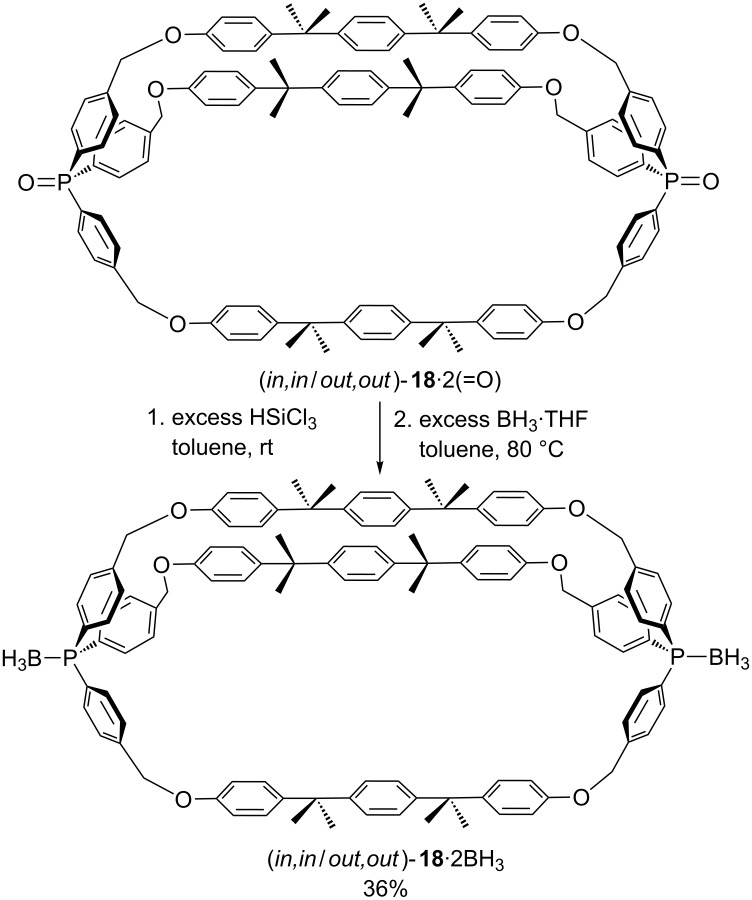
Previously synthesized dibridgehead diphosphine diboranes.

To our knowledge, only one macrocyclic dibridgehead diphosphine diborane has been previously reported, (*in,in/out*,*out*)-**18**·2BH_3_ in Scheme 9 [50–51]. This features triarylphosphorus bridgeheads and *p*-phenylene containing tethers that are long enough to allow rapid homeomorphic isomerization. The precursor **18**·2(=O) was prepared by a threefold Williamson ether synthesis in surprisingly high yields (61% *in*,*in*/*out*,*out* and *in*,*out* combined) [50–51], likely aided by the geminal dialkyl effect associated with the quaternary centers [52].

**Scheme 10:**
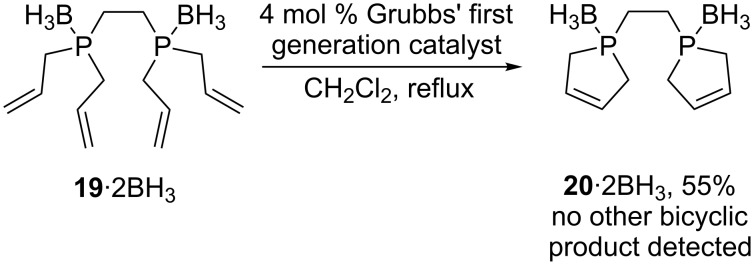
Alkene metathesis of the tetraalkenyldiphosphine diborane **19**·2BH_3_.

Finally, it should be noted that a number of alkene containing phosphine boranes have been employed in metathesis reactions [53–54]. In particular, the tetraalkenyl diphosphine diborane **19**·2BH_3_ in Scheme 10 represents a downsized version of **11**·2BH_3_. A species analogous to **6**·2BH_3_, **20**·2BH_3_, is obtained in much higher yield than any of the products in Scheme 5 [53]. Hence, selectivities can strongly depend upon the lengths of the methylene segments in the precursor.

## Conclusion

In conclusion, this work constitutes a further installment in the evolution of synthetic strategies for dibridgehead diphosphorus compounds that employ alkene metathesis. The new approaches (Scheme 2; Scheme 3 and Scheme 5) lack metal templates, which differentiates them from the routes presented in Scheme 1. However, neither is competitive with Scheme 1, despite eliminating the requirement for stoichiometric amounts of precious metals. Furthermore, preassembling a diphosphine diborane substrate per Scheme 3 and Scheme 5 is not competitive with the "shotgun" approach in Scheme 2, and both routes require comparably demanding preparative column chromatography. Hence, the most promising direction for future research would seem to be templated syntheses via non-precious metals [55]. This remains an area of ongoing investigation in our laboratory and further results will be reported in due course.

## Experimental

**General.** Reactions (except hydrogenations) were conducted under inert atmospheres using standard Schlenk techniques. All chromatography was carried out under aerobic conditions. Additional data are supplied in Supporting Information File 1.

**Metathesis/hydrogenation of H_3_B·P((CH_2_)_6_CH=CH_2_)_3_** (**1**·BH_3_; Scheme 2 [32]). A Schlenk flask was charged with **1**·BH_3_ (1.177 g, 3.110 mmol) [31] and CH_2_Cl_2_ (320 mL; the resulting solution was 0.0097 M in **1**·BH_3_) and cooled to 0 °C. A solution of Grubbs' first generation catalyst (0.077 g, 0.094 mmol, 3 mol %) in CH_2_Cl_2_ (50 mL) was added dropwise via syringe with stirring over 1 h. The cooling bath was removed. After 2 h, additional Grubbs' first generation catalyst was added as a solid (0.051 g, 0.062 mmol, 2 mol %). The flask was fitted with a condenser and the mixture was refluxed overnight, cooled to room temperature, and passed through a SiO_2_ pad (3 cm), which was rinsed with CH_2_Cl_2_. The eluate was concentrated to ca. 20 mL by rotary evaporation, and transferred to a Fischer–Porter bottle. Wilkinson's catalyst (0.086 g, 0.093 mmol, 3 mol %) was added, and the bottle was partially evacuated and charged with hydrogen (5 bar). The sample was kept at 55 ºC for 60 h. The solvent was removed and the residue was placed at the top of a chromatography column (SiO_2_, 3.5 × 36 cm), which was eluted with hexanes/CH_2_Cl_2_ (3:1 to 1:3 v/v) and then CH_2_Cl_2_. Fractions were assayed by TLC, combined where appropriate, and slowly evaporated to dryness in a fume hood. Some fractions (0.091 g total out of the recovered mass of 0.344 g) consisted of unidentified and/or impure products, or oligomers and polymers. Products that could be characterized are as follows (in order of elution).

**H_3_B·P(*n*-C_8_H_17_)_3_** (**4**·BH_3_ [33]; 0.007 g, 0.018 mmol, 1%), colorless oil. Anal. calcd for C_24_H_54_BP (384.47): C, 74.98; H, 14.16; found: C, 74.93; H, 14.02; ^1^H NMR (400 MHz, CDCl_3_) δ 1.53–1.37 (m, 12H, C*H*_2_), 1.33–1.30 (m, 6H, C*H*_2_), 1.26–1.23 (m, 24H, C*H*_2_), 0.83 (t, ^3^*J*_HH_ = 6.9 Hz, 9H, C*H*_3_), 0.47 and 0.19 (br apparent d, 3H, B*H*_3_); ^13^C{^1^H} NMR (101 MHz, CDCl_3_) δ 31.7 (s, *C*H_2_), 31.1 (d, *J*_CP_ = 12.0 Hz, *C*H_2_), 29.0 (s, *C*H_2_), 28.9 (s, *C*H_2_), 22.9 (d, *J*_CP_ = 34.3 Hz, *C*H_2_), 22.50 (s, *C*H_2_), 22.48 (s, *C*H_2_), 14.0 (s, *C*H_3_); ^31^P{^1^H} NMR (162 MHz, CDCl_3_) δ 15.9 and 15.5 (br apparent d); IR (oil film): 2926 (s), 2856 (m), 2366 (m), 1463 (m), 1413 (w), 1378 (w), 1135 (w), 1061 (m), 1034 (w), 807 (w), 764 (w), 722 (m) cm^−1^; MS (EI) [56]: 384 (M^+^, <1%), 370 ([M − BH_3_]^+^, 79%).


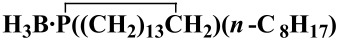
(**5**·BH_3_; 0.090 g, 0.25 mmol, 8%), colorless oil. Anal. calcd for C_22_H_48_BP (354.40): C, 74.56; H, 13.65; found: C, 74.27; H, 13.52; ^1^H NMR (500 MHz, CDCl_3_) δ 1.62–1.19 (m, 42H, C*H*_2_), 0.86 (t, 3H, ^3^*J*_HH_ = 7.0 Hz, C*H*_3_), 0.48 and 0.26 (br apparent d, 3H, B*H*_3_); ^13^C{^1^H} NMR (126 MHz, CDCl_3_) δ 31.7 (s, *C*H_2_), 31.2 (d, *J*_CP_ = 12.6 Hz, *C*H_2_), 29.03 (s, *C*H_2_), 29.01 (s, *C*H_2_), 28.9 (d, *J*_CP_ = 11.1 Hz, 2*C*H_2_), 26.7 (s, 2*C*H_2_), 26.53 (s, 2*C*H_2_), 26.48 (s, 2*C*H_2_), 26.1 (s, 2*C*H_2_), 23.8 (d, *J*_CP_ = 35.4 Hz, *C*H_2_), 22.57 (d, *J*_CP_ = 1.2 Hz, 2*C*H_2_), 22.55 (s, *C*H_2_), 22.3 (d, *J*_CP_ = 33.6 Hz, *C*H_2_), 21.2 (d, *J*_CP_ = 3.3 Hz, 2*C*H_2_), 14.0 (s, *C*H_3_); ^31^P {^1^H} NMR (202 MHz, CDCl_3_) δ 15.6 and 15.2 (br apparent d); IR (oil film): 2926 (s), 2856 (m), 2366 (m), 1459 (m), 1417 (w), 1135 (w), 1061 (m), 811 (m), 760 (m), 722 (m) cm^−1^; MS (EI) [56]: 340 ([M − BH_3_]^+^, 93%), 228 ([M − BH_3_ − C_8_H_17_ + 1]^+^, 100%).

***in,out*-H_3_B·P((CH_2_)_14_)_3_P·BH_3_** (*in*,*out*-**2**·2BH_3_; 039 g, 0.057 mmol, 4%), colorless oil. Anal. calcd for C_42_H_90_B_2_P_2_ (678.73): C, 74.32; H, 13.37; found: C, 73.86; H, 13.49; ^1^H NMR (500 MHz, CDCl_3_) δ 1.56–1.51 (m, 12H, PC*H*_2_), 1.49–1.42 (m, 12H, C*H*_2_), 1.39–1.33 (m, 12H, C*H*_2_), 1.31–1.21 (m, 48H, C*H*_2_), 0.45 and 0.27 (br apparent d, 6H, B*H*_3_); ^13^C{^1^H} NMR (126 MHz, CDCl_3_) δ 30.5 (d, *J*_CP_ = 11.3 Hz, *C*H_2_), 28.35 (s, *C*H_2_), 28.28 (s, *C*H_2_), 28.2 (s, *C*H_2_), 28.1 (s, *C*H_2_), 23.0 (d, *J*_CP_ = 34.3 Hz, *C*H_2_), 22.2 (d, *J*_CP_ = 1.9 Hz, *C*H_2_); ^31^P{^1^H} NMR (202 MHz, CDCl_3_) δ 15.6 and 15.4 (br apparent d); IR (oil film): 2926 (s), 2853 (m), 2366 (w), 1459 (w), 1413 (w), 1135 (w), 1061 (m), 803 (w), 722 (w) cm^−1^; MS (MALDI^+^, THAP) [56]: 651.6 ([M – 2BH_3_ + 1]^+^, 100%).


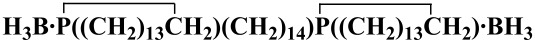
 (**6**·2BH_3_; 0.101 g, 0.149 mmol, 10%), white solid, mp 96 °C (capillary). Anal. calcd for C_42_H_90_B_2_P_2_ (678.73): C, 74.32; H, 13.37; found: C, 73.92; H, 13.47. The identity of this compound, which has been independently synthesized, has been confirmed crystallographically [6]. ^1^H NMR (500 MHz, CDCl_3_) δ 1.65–1.14 (br m, 84H, C*H*_2_), 0.49 and 0.26 (br apparent d, 6H, B*H*_3_); ^13^C{^1^H} NMR (126 MHz, CDCl_3_) δ 31.3 (d, *J*_CP_ = 12.6 Hz, *C*H_2_), 29.54 (s, *C*H_2_), 29.53 (s, *C*H_2_), 29.4 (s, *C*H_2_), 29.1 (s, *C*H_2_), 29.0 (d, *J*_CP_ = 11.1 Hz, 2*C*H_2_), 26.8 (s, 2*C*H_2_), 26.6 (s, 2*C*H_2_), 26.5 (s, 2*C*H_2_), 26.1 (s, 2*C*H_2_), 23.8 (d, *J*_CP_ = 35.3 Hz, *C*H_2_), 22.6 (d, *J*_CP_ = 1.0 Hz, *C*H_2_), 22.3 (d, *J*_CP_ = 33.5 Hz, 2*C*H_2_), 21.2 (d, *J*_CP_ = 3.3 Hz, 2*C*H_2_); ^31^P {^1^H} NMR (202 MHz, CDCl_3_) δ 15.6 and 15.2 (br apparent d); IR (powder film): 2922 (s), 2853 (m), 2366 (m), 1459 (m), 1417 (w), 1135 (w), 1061 (m), 791 (w), 722 (m) cm^−1^; MS (EI) [56]: 678 (M^+^, 9%), 665 ([M − BH_3_]^+^, 100%), 652 ([M − 2BH_3_ + 1]^+^, 72%).

**(*in,in/out,out*)-H_3_B·P((CH_2_)_14_)_3_P·BH_3_** ((*in*,*in*/*out*,*out*)-**2**·2BH_3_; 0.016 g, 0.024 mmol, 2%), colorless oil that solidified to give a white powder, mp 112 °C. Anal. calcd for C_42_H_90_B_2_P_2_ (678.73): C, 74.32; H, 13.37; found: C, 74.71; H, 13.34; ^1^H NMR (500 MHz, CDCl_3_) δ 1.55–1.50 (m, 12H, C*H*_2_), 1.47–1.39 (m, 12H, C*H*_2_), 1.37–1.32 (m, 12H, C*H*_2_), 1.29–1.21 (m, 48H, C*H*_2_), 0.38 and 0.26 (br apparent d, 6H, B*H*_3_); ^13^C{^1^H} NMR (126 MHz, CDCl_3_) δ 30.6 (d, *J*_CP_ = 12.1 Hz, *C*H_2_), 29.23 (s, *C*H_2_), 29.17 (s, *C*H_2_), 28.9 (s, *C*H_2_), 28.4 (s, *C*H_2_), 22.5 (d, *J*_CP_ = 34.1 Hz, *C*H_2_), 22.1 (d, *J*_CP_ = 2.7 Hz, *C*H_2_); ^31^P{^1^H} NMR (202 MHz, CDCl_3_) δ 14.9 and 14.7 (br apparent d); IR (powder film): 2922 (s), 2853 (s), 2366 (m), 1467 (m), 1413 (w), 1131 (w), 1061 (m), 807 (w), 760 (w), 718 (m) cm^−1^; MS (MALDI^+^, THAP) [56]: 702.0 ([M + Na]^+^, 98%), 666.0 ([M − BH_3_ + 1]^+^, 100%).

**Metathesis/hydrogenation of (H_2_C=CH(CH_2_)_6_)_2_(H_3_B)P((CH_2_)_14_)P(BH_3_)((CH_2_)_6_CH=CH_2_)_2_** (**11**·2BH_3_; Scheme 5 [32]). Diphosphine diborane **11**·2BH_3_ (1.222 g, 1.672 mmol), CH_2_Cl_2_ (1700 mL; the resulting solution was 0.0010 M in **11**·2BH_3_), Grubbs' first generation catalyst (0.069 g, 0.083 mmol, 5 mol %), Wilkinson's catalyst (0.046 g, 0.050 mmol, ca. 3 mol %), and H_2_ were combined in a procedure analogous to that used for **1**·BH_3_. An identical work-up gave *in,out*-**2**·2BH_3_ (0.072 g, 0.106 mmol, 6%, minor impurities evident by ^13^C{^1^H} NMR), **6**·2BH_3_ (0.056 g, 0.083 mmol, 5%, minor impurities evident by ^13^C{^1^H} NMR), and (*in,in*/*out*,*out*)-**2**·2BH_3_ (0.075 g, 0.111 mmol, 7%), along with several fractions consisting of unidentified and/or impure products, or oligomers and polymers. Spectroscopic data for *in,out*-**2**·2BH_3_, (*in,in*/*out*,*out*)-**2**·2BH_3_, and **6**·2BH_3_ matched those reported above.

## Supporting Information

File 1 Additional experimental data.
