# Supplementary material for: Non-metal-templated approaches to bis(borane) derivatives of macrocyclic dibridgehead diphosphines via alkene metathesis
Source: Beilstein J Org Chem. 2018 Sep 7;14:2354–65. doi: 10.3762/bjoc.14.211 (PMC6142775; doi:10.3762/bjoc.14.211)
Supplement: File 1 — Additional experimental data. [file Beilstein_J_Org_Chem-14-2354-s001.pdf]

Supporting Information

for

Non-metal-templated approaches to bis(borane) derivatives of macrocyclic dibridgehead diphosphines via alkene metathesis

Tobias Fiedler[1,2], Michał Barbasiewicz[2,3], Michael Stollenz[1,4] and John A. Gladysz*[1,2]

Address: [1]Department of Chemistry, Texas A&M University, PO Box 30012, College Station, Texas 77842-3012, USA, [2]Institut für Organische Chemie and Interdisciplinary Center for Molecular Materials, Friedrich-Alexander-Universität Erlangen-Nürnberg, Henkestraße 42, 91054 Erlangen, Germany, [3]Present address: Faculty of Chemistry, University of Warsaw, Pasteura 1, 02-093 Warszawa, Poland and [4]Present address: Department of Chemistry and Biochemistry, Kennesaw State University, 370 Paulding Building NW, MD#1203, Kennesaw, Georgia 30144, USA

Email: John A. Gladysz - gladysz@mail.chem.tamu.edu

*Corresponding author

Additional experimental data

Experimental (continued)

General (continued). Solvents were treated as follows: THF and Et_2O, distilled from Na/benzophenone or dried using a solvent dispensing system (Seca Solvent System); CH_2Cl_2, distilled from CaH_2 or dried using a solvent dispensing system (Seca Solvent System); ethyl acetate, Et_2O, hexanes, CH_2Cl_2 (for aerobic manipulations), distilled by rotary evaporation or used as received (ACS grade); C_6D_6, $CDCl_3$, CD_3OD (3 × deutero GmbH or Cambridge Isotope Laboratories), used as received. The 1,14-tetradecanedioic acid (97%, Astatech), *N*-methylmorpholine (99%, Acros), ethyl chloroformate (99%, Acros), $NaBH_4$ (98%, Acros), CBr_4 (99%, Aldrich), PPh_3 (99%, Acros), $P(OEt)_3$ (98%, Acros), $LiAlH_4$ (99%, Acros or Aldrich), $H_3B{\cdot}SMe_2$ (2.0 M in THF, Acros), *n*-BuLi (2.5 M in hexanes, Acros), triphosgene (98%, Alfa Aesar), Grubbs' first generation catalyst ($(Cy_3P)_2Cl_2Ru{=}CHPh$; Aldrich, 97%), Wilkinson's catalyst ($(Ph_3P)_3RhCl$; 97%, Strem), SiO_2, neutral Al_2O_3 (2 × Macherey-Nagel), and $MgSO_4$ (Acros) were used as received.

NMR spectra were obtained on Bruker (Avance 300/400; 300/400 MHz), Jeol (JEOL GX400 or JEOL EX400; 400 MHz), or Varian (Inova 300, Mercury 300; 300 MHz, or NMRS 500; 500 MHz) spectrometers and referenced as follows: (δ, ppm): ^1H NMR, residual C_6D_5H (7.15), $CHCl_3$ (7.26), or CD_2HOD (3.31); $^{13}C\{^1H\}$ NMR, C_6D_6 (128.0), $CDCl_3$ (77.0), or CD_3-OD (49.2); $^{31}P\{^1H\}$ NMR, external H_3PO_4 (0.00). Mass spectra were obtained using Micromass Zabspec (EI, FAB), Shimadzu Biotech Axima Confidence (MALDI-TOF MS), Applied Biosystem STR Voyager (MALDI-TOF MS), or PE Sciex QStar (APCI) instruments. DSC and TGA data were recorded with a Mettler-Toledo DSC821 instrument. IR spectra were recorded on ASI React-IR 1000, Thermo SCIENTIFIC Nicolet IR100 FT-IR, or Shimadzu IRAffinity-1 (equipped with a Pike MIRacle ATR system) spectrometers. Microanalyses were conducted on a Carlo Erba EA1110 instrument (in house) or by Atlantic Microlab.

$HO(CH_2)_{14}OH$ (7) [S1,S2]**.** A Schlenk flask was charged with 1,14-tetradecanedioic acid (5.40 g, 20.90 mmol) and THF (220 mL) and cooled to –5 °C. *N*-Methyl morpholine (4.58 mL, 41.80 mmol) and then ethyl chloroformate (4.22 mL, 43.99 mmol) were added with stirring.

After 10 min, the white precipitate was removed by filtration and the filtrate cooled in an ice bath. A solution of $NaBH_4$ (2.52 g, 66.7 mmol) in water (25 mL) was added with stirring. After gas evolution ceased, water (300 mL) was added and the aqueous phase extracted with ethyl acetate (3 × 100 mL). The combined organic layers were dried ($MgSO_4$) and filtered. The solvents were removed by rotary evaporation to give **7** (4.66 g, 20.2 mmol, 96%) as a white powder, mp 85 °C (capillary); lit: 85-87 °C [S1].

NMR (CD_3OD, δ/ppm): **^{1}H** (300 MHz) 3.54 (t, 4H, $^3J_{HH}$ = 6.6 Hz, CH_2OH), 1.58-1.46 (m, 4H, CH_2), 1.40-1.25 (m, 20H, CH_2); **^{13}C{^{1}H}** (75 MHz) 63.1 (s, CH$_2$OH), 33.7 (s, CH$_2$), 30.83 (s, CH$_2$), 30.80 (s, CH$_2$), 30.7 (s, CH$_2$), 27.0 (s, CH$_2$). **IR** (cm^{-1}, powder film): 3408 (m), 3341 (m), 3210 (m), 2920 (s), 2849 (s), 1462 (s), 1356 (m), 1196 (w), 1121 (s), 1018 (s), 972 (m), 727 (m). **MS** (FAB, 3-NBA) [S3]: 231 ([**M** + 1]$^+$, 65%), 154 (100%).

$Br(CH_2)_{14}Br$ (8) [S2,S4]**.** A Schlenk flask was charged with **7** (5.00 g, 21.70 mmol), CBr_4 (21.59 g, 65.10 mmol), and THF (250 mL). Then PPh_3 (17.80 g, 65.10 mmol) was added in portions with stirring. After 16 h, water (200 mL) was added and the aqueous phase extracted with CH_2Cl_2 (3 × 200 mL). The combined organic layers were dried ($MgSO_4$) and filtered. The solvents were removed by rotary evaporation. Hexanes (500 mL) were added to the brown residue, and the precipitate ($O{=}PPh_3$) removed by filtration. The solvent was removed from the filtrate by rotary evaporation and the residue chromatographed (SiO_2 column, 4 cm × 30 cm, hexanes). The solvent was removed from the product containing fractions to give **8** (6.57 g, 18.45 mmol, 85%) as a white powder, mp 51 °C; lit: 47.5-48.0 °C [S5].

NMR (C_6D_6, δ/ppm): **^{1}H** (300 MHz) 2.97 (t, 4H, $^3J_{HH}$ = 6.8 Hz, CH_2Br), 1.57-1.46 (m, 4H, CH_2CH$_2$Br), 1.31-1.03 (m, 20H, CH_2); **^{13}C{^{1}H}** (75 MHz) 33.7 (s, CH$_2$), 33.0 (s, CH$_2$), 30.0 (s, CH$_2$), 29.9 (s, CH$_2$), 29.8 (s, CH$_2$), 29.0 (s, CH$_2$), 28.4 (s, CH$_2$). **IR** (cm^{-1}, powder film): 2914 (s), 2851 (s), 1472 (s), 1340 (w), 1308 (w), 1260 (m), 1215 (m), 1196 (w), 1030 (w), 718 (m). **MS** (APCI) [S3]: 377 ([**M** + 1]$^+$, 50%), 360 (100%).

$(EtO)_2(O{=})P((CH_2)_{14})P({=}O)(OEt)_2$ (9) [S2,S6]**.** A Schlenk flask was charged with $P(OEt)_3$ (5.83 g, 35.10 mmol) and **8** (5.00 g, (14.04 mmol), and fitted with a distillation head.

The mixture was stirred at 150 °C overnight, and monitored by TLC (hexanes). In cases where **8** was not completely consumed, an additional portion of $P(OEt)_3$ was added and stirring continued until full conversion. Excess $P(OEt)_3$ was removed by distillation under oil pump vacuum. After cooling, **9** (6.33 g, 13.45 mmol, 96%) was obtained as a colorless liquid. Anal. calcd (%) for $C_{22}H_{48}O_6P_2$ (470.56): C 56.15, H 10.28; found C 56.28, H 9.98.

NMR (C_6D_6, δ/ppm): ^{1}**H** (300 MHz) 4.03-3.87 (m, 8H, OCH_2), 1.72-1.56 (m, 8H, CH_2), 1.30-1.16 (m, 20H, CH_2), 1.07 (t, 12H, $^3J_{HH}$ = 7.1 Hz, CH_3); ^{13}**C{^{1}H}** (75 MHz) 61.0 (d, $^2J_{CP}$ = 6.3 Hz, OCH_2), 30.9 (d, J_{CP} = 16.0 Hz, CH_2), 30.04 (s, CH_2), 30.01 (s, CH_2), 29.8 (s, CH_2), 29.5 (s, CH_2), 27.4 (s, CH_2) 25.5 (s, CH_2), 23.0 (d, J_{CP} = 5.1 Hz, CH_2), 16.6 (d, $^3J_{CP}$ = 5.6 Hz, CH_3); ^{31}**P{^{1}H}** (121 MHz) 32.4 (s). **IR** (cm^{-1}, oil film): 2984 (w), 2926 (m), 2856 (w), 1467 (w), 1393 (w), 1243 (m), 1166 (w), 1096 (w), 1054 (m), 1023 (s), 953 (s), 787 (m), 722 (w). **MS** (APCI) [S3]: 471 ([**M** + 1]$^+$, 100%).

$H_2P((CH_2)_{14})PH_2$ (10) [S2]**.** A Schlenk flask was fitted with a dropping funnel and charged with $LiAlH_4$ (1.31 g, 32.97 mmol) and Et_2O (100 mL). The suspension was cooled to 0 °C and a solution of **9** (5.00 g, 10.63 mmol) in Et_2O (10 mL) was added dropwise with stirring over 30 min. The cooling bath was removed. After 16 h, the suspension was cooled to 0 °C and water (ca. 5 mL) was added dropwise until gas evolution ceased. The white suspension was transferred to a flask with $MgSO_4$ and then filtered through a short pad of Al_2O_3, which was washed with Et_2O. The solvent was removed from the filtrate by oil pump vacuum to give **10** (2.70 g, 10.29 mmol, 97%) as a waxy white solid, mp 30 °C (sealed capillary).

NMR (C_6D_6, δ/ppm): ^{1}**H** (300 MHz) 2.65 (dm, 4H, $^1J_{HP}$ = 190.0 Hz, PH_2), 1.40-1.12 (m, 28H, CH_2); ^{13}**C{^{1}H}** (75 MHz) 33.3 (d, J_{CP} = 3.5 Hz, CH_2), 30.9 (d, J_{CP} = 5.7 Hz, CH_2), 30.13 (s, CH_2), 30.10 (s, CH_2), 30.0 (s, CH_2), 29.6 (s, CH_2), 14.0 (d, J_{CP} = 8.1 Hz, CH_2); ^{31}**P{^{1}H}** (121 MHz) −137.8 (s).

$H_2(H_3B)P((CH_2)_{14})P(BH_3)H_2$ (10·2BH$_3$) [S2]**.** A Schlenk flask was charged with **10** (0.250 g, 0.953 mmol) and THF (15 mL), and cooled to 0 °C. Then $H_3B{\cdot}SMe_2$ (2.0 M in THF, 0.96 mL, 1.92 mmol) was added dropwise with stirring over 20 min. The cooling bath was

removed. After 16 h, a $^{31}P\{^1H\}$ NMR spectrum showed the consumption of **10**. Volatiles were removed by oil pump vacuum to give **10**·$2BH_3$ (0.240 g, 0.828 mmol, 87%) as a white solid, mp 55 °C. **DSC** ($T_i/T_e/T_p/T_c/T_f$) [S7]: 37.5/44.4/52.7/55.8/58.2 °C (endotherm). **TGA**: onset of mass loss, 90.4 °C. Anal. calcd (%) for $C_{14}H_{38}B_2P_2$ (290.02): C 57.98, H 13.21; found C 57.23, H 13.21 [S8].

NMR (C_6D_6, δ/ppm): ^{1}**H** (300 MHz) 3.67 (dm, 4H, $^1J_{PH}$ = 358.0 Hz, PH_2), 1.41 (overlapping br qm, $^1J_{BH}$ = 96.9 Hz, 6H, BH_3) [S9], 1.43-0.78 (overlapping m, 28H, CH_2); ^{13}**C{^{1}H}** (75 MHz) 30.4 (d, J_{CP} = 10.7 Hz, CH_2), 30.1 (s, CH_2), 30.0 (s, CH_2), 29.7 (s, CH_2), 29.2 (s, CH_2), 26.5 (d, J_{CP} = 5.1 Hz, CH_2), 16.4 (d, J_{CP} = 35.1 Hz, CH_2); ^{31}**P{^{1}H}** (121 MHz) −39.3 and −39.6 (br apparent d). **IR** (cm^{-1}, powder film): 2918 (s), 2849 (s), 2405 (s), 2378 (s), 2351 (m), 1472 (m), 1464 (m), 1134 (w), 1096 (m), 1049 (s), 993 (m), 902 (s), 787 (m), 721 (m). **MS** (FAB, 3-NBA) [S3]: 291 ([**M** □ 1]$^+$, 20%), 275 ([**M** – BH_3 –1]$^+$, 50%), 261 ([**M** – $2BH_3$ – 1]$^+$, 136 (100%).

$(H_2C{=}CH(CH_2)_6)_2P((CH_2)_{14})P((CH_2)_6CH{=}CH_2)_2$ (11) [S2]. A Schlenk flask was charged with **10** (0.930 g, 3.545 mmol) and THF (20 mL) and cooled to 0 °C. Then *n*-BuLi (2.5 M in hexanes, 5.81 mL, 14.54 mmol) was added dropwise with stirring over 15 min. Then Br(C-$H_2)_6CH{=}CH_2$ (2.710 g, 14.18 mmol) [S10] was similarly added over 10 min. The cooling bath was removed. After 3 h, the solvents were removed by oil pump vacuum and the residue filtered through a short pad of Al_2O_3 (5 cm) with CH_2Cl_2. The solvent was removed from the filtrate by oil pump vacuum to give **11** (1.795 g, 2.553 mmol, 72%) as a colorless oil.

NMR (C_6D_6, δ/ppm): ^{1}**H** (300 MHz) 5.78 (ddt, 4H, $^3J_{HHtrans}$ = 17.0 Hz, $^3J_{HHcis}$ = 10.1 Hz, $^3J_{HH}$ = 6.7 Hz, CH=), 5.04 (br d, 4H, $^3J_{HHtrans}$ = 17.2 Hz, 4H, =CH_EH_Z), 4.99 (br d, 4H, $^3J_{HHcis}$ = 10.2 Hz, 6H, =CH_EH_Z), 2.05-1.93 (m, 8H, CH_2CH=), 1.62-1.18 (br m, 68H, CH_2); ^{13}**C{^{1}H}** (75 MHz) 139.1 (s, CH=), 114.5 (s, =CH_2), 34.2 (s, CH_2CH=), 32.0 (d, J_{CP} = 10.5 Hz, CH_2), 31.8 (d, J_{CP} = 10.6 Hz, 2CH_2), 30.2 (s, CH_2), 30.1 (s, CH_2), 30.0 (s, CH_2), 29.9 (s, CH_2), 29.3 (2CH_2), 29.2 (s, 2CH_2), 28.22 (d, J_{CP} = 14.1 Hz, CH_2), 28.19 (d, J_{CP} = 14.2 Hz, 2CH_2), 26.6 (d, J_{CP} = 13.6 Hz, CH_2), 26.5 (d, J_{CP} = 13.5 Hz, 2CH_2); ^{31}**P{^{1}H}** (121 MHz) –30.9 (s).

$(H_2C{=}CH(CH_2)_6)_2(H_3B)P((CH_2)_{14})P(BH_3)((CH_2)_6CH{=}CH_2)_2$ (11·2BH$_3$) [S2]. A Schlenk flask was charged with **10**·2BH$_3$ (1.05 g, 3.62 mmol) and THF (15 mL), and cooled to –78 °C. Then *n*-BuLi (2.5 M in hexanes, 6.4 mL, 16.0 mmol) was added with stirring. After 30 min, Br(CH$_2$)$_6$CH=CH$_2$ (2.80 g, 14.65 mmol) [S10] was added to the pale yellow solution over 10 min. After 1 h, the cooling bath was removed. After 16 h, a ^{31}P{^{1}H} NMR spectrum showed the consumption of **10**·2BH$_3$, and water (10 mL) was added. The aqueous phase was extracted with Et$_2$O (4 × 10 mL), and the combined organic layers dried (MgSO$_4$) and filtered. The solvents were removed by rotary evaporation and the residue chromatographed (SiO$_2$ column, 4 cm × 30 cm, hexanes). The solvent was removed from the product containing fractions by rotary evaporation to give **11**·2BH$_3$ (0.476 g, 0.652 mmol, 18%) as a colorless oil. Anal. calcd (%) for $C_{46}H_{94}B_2P_2$ (730.81): C 75.60, H 12.96; found C 75.50, H 12.98.

NMR (C$_6$D$_6$, δ/ppm): **^{1}H** (500 MHz) 5.78 (ddt, 4H, $^3J_{HHtrans}$ = 16.9 Hz, $^3J_{HHcis}$ = 10.2 Hz, $^3J_{HH}$ = 6.7 Hz, C*H*=), 5.05 (br d, 4H, $^3J_{HHtrans}$ = 17.1 Hz, =CH$_E$*H*$_Z$), 5.01 (br d, 4H, $^3J_{HHcis}$ = 10.1 Hz, 4H, =C*H*$_E$H$_Z$), 2.00-1.95 (m, 8H, C*H*$_2$CH=), 1.51-1.13 (br m, 74H, C*H*$_2$ and B*H*$_3$); **^{13}C{^{1}H}** (126 MHz) 139.0 (s, *C*H=), 114.7 (s, =*C*H$_2$), 34.1 (s, *C*H$_2$CH=), 31.6 (d, J_{CP} = 11.9 Hz, *C*H$_2$), 31.4 (d, J_{CP} = 11.9 Hz, 2*C*H$_2$), 30.11 (s, *C*H$_2$), 30.07 (s, *C*H$_2$), 29.9 (s, *C*H$_2$), 29.5 (s, *C*H$_2$), 29.2 (s, 2*C*H$_2$), 28.9 (s, 2*C*H$_2$), 23.62 (d, J_{CP} = 33.3 Hz, *C*H$_2$), 23.60 (d, J_{CP} = 33.3 Hz, 2*C*H$_2$), 23.1 (d, J_{CP} = 1.8 Hz, *C*H$_2$), 23.0 (d, J_{CP} = 1.8 Hz, 2*C*H$_2$); **^{31}P{^{1}H}** (202 MHz) 15.8 and 15.5 (br apparent d). **IR** (cm^{-1}, oil film): 3074 (w), 2918 (s), 2853 (s), 2365 (m), 2334 (m), 1639 (m), 1462 (m), 1414 (m), 1132 (w), 1057 (m), 993 (m), 908 (s), 802 (m), 737 (s). **MS** (APCI) [S3]: 730 ([**M** – 1]$^+$, 30%), 718 ([**M** – BH$_3$ □ 1]$^+$, 60%), 704 ([**M** – 2BH$_3$ □ 1]$^+$, 100%).

$Cl_2P((CH_2)_{14})PCl_2$ (12) [S2]. A Schlenk flask was charged with **10** (4.010 g, 15.28 mmol) and CH$_2$Cl$_2$ (150 mL) and cooled to –40 °C. A solution of triphosgene (6.589 g, 22.20 mmol) in CH$_2$Cl$_2$ (50 mL) was added dropwise with stirring over 40 min. The cooling bath was removed. After 16 h, the solvent was removed by oil pump vacuum. The oily residue was stirred at 50 °C for 1 h under oil pump vacuum to remove residual triphosgene, giving **12** (5.747 g, 14.36 mmol, 94%) as a pale yellow oil.

NMR ($CDCl_3$, δ/ppm): **^{1}H** (500 MHz) 2.36-2.28 (m, 4H, PCH_2), 1.72-1.64 (m, 4H, PCH_2CH_2), 1.48-1.41 (m, 4H, $PCH_2CH_2CH_2$), 1.37-1.23 (br m, 16H, CH_2); **^{13}C{^{1}H}** (126 MHz) 43.3 (d, $^1J_{CP}$ = 44.0 Hz, PCH_2), 30.3 (s, CH_2), 30.2 (s, CH_2), 29.6 (s, CH_2), 29.5 (s, CH_2), 29.2 (d, J_{CP} = 20.8 Hz, CH_2), 23.1 (d, $^2J_{CP}$ = 13.5 Hz, PCH_2CH_2); **^{31}P{^{1}H}** (202 MHz) 196.5 (s).

References

[S1] Nguyen, T. B.; Castanet, A.-S.; Nguyen, T.-H.; Nguyen, K. P. P.; Bardeau, J.-F.; Gibaud, A.; Mortier, J. *Tetrahedron* **2006**, *62*, 647-651.

[S2] Fiedler, T. Syntheses of Gyroscope-like Osmium Complexes and Cage-like Diphosphines. Doctoral dissertation, Friedrich-Alexander-University, Erlangen, Germany, 2011.

[S3] The most intense peak of the isotope envelope is given; *m*/*z* (relative intensity, %). Matrices used: 3-NBA (3-nitrobenzyl alcohol), THAP (2,4,6-trihydroxyacetophenone).

[S4] Methodology used to prepare **8**: Soomro, S. A.; Benmouna R.; Berger, R.; Meier, H. *Eur. J. Org. Chem.* **2005**, 3586-3593.

[S5] Woolford, R. G. *Can. J. Chem.* **1962**, *40*, 1846-1850.

[S6] For a related Arbuzov reaction of **8**, see the supporting information of HailuTaffa, D.; Kathiresan, M.; Walder, L. *Langmuir* **2009**, *25*, 5371-5379.

[S7] Cammenga, H. K.; Epple, M. *Angew. Chem., Int. Ed. Engl.* **1995**, *34*, 1171-1187; *Angew. Chem.* **1995**, *107*, 1284-1301. The T_e values best represent the temperature of phase transition or endotherm. DSC measurements were generally not continued above the temperature of initial mass loss (TGA).

[S8] This microanalysis only marginally agrees with the empirical formula, but is nonetheless reported as the best obtained to date. The NMR spectra indicate high purities (≥98%). See also Gabbaï, F. P.; Chirik, P. J.; Fogg, D. E.; Meyer, K.; Mindiola, D. J.; Schafer, L. L.; You, S.-L. *Organometallics* **2016**, *35*, 3255-3256.

[S9] The coupling constant was derived from two unobscured peaks of the quartet.

[S10] Roux, M.-C.; Paugam, R.; Rousseau, G. *J. Org. Chem.* **2001**, *66*, 4304-4310.
